# Psychiatric Morbidity: A Retrospective Study From a Tertiary Care Center

**DOI:** 10.7759/cureus.36687

**Published:** 2023-03-26

**Authors:** Sanjay Prasad, Bhupendra K Rohit, Abhijit Das, Vishal Choubey

**Affiliations:** 1 Psychiatry, Government Bundelkhand Medical College, Sagar, IND; 2 Community Medicine, Government Bundelkhand Medical College, Sagar, IND

**Keywords:** disease pattern, mental health, tertiary care centers, psychiatric illness, psychiatric morbidity

## Abstract

Background

It is crucial to monitor the psychiatric morbidity patterns of patients to comprehend the burden and trends of mental illness, as well as to create targeted prevention and intervention strategies. Due to the significant regional differences in mental illness, the current study assessed the psychiatric morbidity pattern from a tertiary care center in Central India.

Methods

We conducted this retrospective record-based study using data from the outpatient department register of the Psychiatry Department of Government Bundelkhand Medical College, Sagar, Madhya Pradesh, India. All records from January to December 2022 were included, while duplicate and incomplete records were excluded. Data from 2005 cases were finalized for analysis after considering inclusion and exclusion criteria. Data abstraction was done for age, gender, marital status, family history of any psychiatric disorder, and diagnosis (according to ICD-10) from the records. Data analysis was conducted using SPSS Version 26.0 (IBM Corp., Armonk, NY). Quantitative data were presented as means ± standard deviation (SD), whereas qualitative data were presented as frequency and percentages. The chi-square test was applied to determine the association, and p-values <0.05 were considered significant.

Results

The mean age of the patients was 37.2±16.9 years, where the youngest patient was of four years of age and the eldest was 85 years of age. Most patients were males (50.6%), married (61.1%), and from rural areas (71.8%). Mood (affective) disorder (32.4%) was the most common, followed by schizophrenia, schizotypal and delusional disorders (20.0%), and neurotic, stress-related, and somatoform disorders (17.4%). Organic mental disorders and substance use disorders were more common in unmarried individuals and males. Females had higher rates of mood disorders and somatoform disorders, with varying age distributions. Adult personality disorder and mental retardation had equal frequencies among males and females, with different age distributions. Hyperkinetic disorder was more common in males, while headache syndrome was more common in females. Psychiatric disorders were more prevalent in the urban population, except for substance abuse and hyperkinetic disorder.

Conclusion

Our study highlights the types of psychiatric disorders among patients at a tertiary care center, aiding clinicians in improving care and emphasizing early detection and treatment of mental illnesses.

## Introduction

Mental health and well-being have been recognized as crucial factors for an individual’s overall health and quality of life. The World Health Organization (WHO) released a landmark report titled “Mental Health: New Hope, New Understanding” in 2001, which highlighted the importance of addressing mental health issues as a means of reducing the total disease burden [1]. Since then, several countries, including India, have significantly improved their mental healthcare systems.

India launched its National Mental Health Policy in 2014 and a revised Mental Healthcare Act in 2017, with the objective of providing equitable, affordable, and universal access to mental health care [2,3]. The country’s efforts to improve mental healthcare are particularly important given that a global burden of disease study on mental health reported that one in seven Indians were affected by mental disorders of varying severity in 2017 [4]. Furthermore, the proportional contribution of mental disorders to the total disease burden in India has almost doubled since 1990, underscoring the urgency of addressing mental health issues in the country [4].

The National Mental Health Survey found that in Madhya Pradesh, the prevalence of common mental disorders was 13.5%, severe mental disorders 0.4%, alcohol use disorder 10.3%, depressive disorder 1.4%, and high suicidal risk 0.8% [5]. However, no documented data at the district level or for Sagar district, Madhya Pradesh, India, were available. The lack of data at the district level makes it difficult to identify and address specific mental health issues in the region. Additionally, no research study has been conducted on mental health in the Sagar district, highlighting the need for further investigation and intervention.

It is essential to recognize the need for mental health resources in all regions of a country. Addressing mental health issues at the local level can provide individuals with the care they need and improve overall well-being in the region. By investing in mental health resources and improving data collection methods, India can continue to make progress in addressing mental health issues and reducing the total disease burden.

Government Bundelkhand Medical College is the only medical college in the Sagar district. This tertiary care center caters to the population from both urban and rural areas of the Sagar district and nearby districts. Therefore, this study aimed to find the pattern of psychiatric disorders among patients attending this tertiary care hospital’s psychiatry outpatient department (OPD).

## Materials and methods

Study design, setting, and duration

We conducted a retrospective record-based study between January 2023 and February 2023 after getting approval from the Institutional Ethics Committee, Government Bundelkhand Medical College, Sagar. Data were collected from the OPD register of the Psychiatry Department of Government Bundelkhand Medical College from January 2022 to December 2022.

Inclusion and exclusion criteria

All records for the one year (January to December 2022) were included, while duplicate data (of the same patient/follow-up cases) and incomplete records were excluded.

Sample size and sampling

A total of 4,745 entries were found in the psychiatric OPD register between January and December 2022. Among them, 2,595 cases were follow-up cases, and 145 entries had incomplete data. Data from 2005 cases were finalized for analysis. Nonprobability sampling was done for this study purpose.

Study variables and data analysis

Data were abstracted in a data abstraction sheet with the help of EpiInfo (version 7) software. Data abstraction was done for the date of consultation, age, gender, marital status, family history of any psychiatric disorder, and diagnosis (according to ICD-10) from the records for further analysis. Data analysis was conducted using Statistical Package for Social Sciences (SPSS) software Version 26 (IBM Corp., Armonk, NY). Quantitative data were presented as the means ± standard deviation (SD), whereas qualitative data were presented as frequency and percentages. The chi-square test was applied to find out the association of different variables with psychiatric morbidity, and p-values < 0.05 were considered significant.

## Results

Patient’s characteristics

The mean age of the patients was 37.2±16.9 years, where the youngest patient was of four years of age and the eldest was 85 years of age. Table 1 describes the characteristics of the patients.

**Table 1:** Characteristics of patients (N=2,005)

Variables	Frequency	Percentage
Gender		
Female	990	49.4%
Male	1,015	50.6%
Age group		
<20 years	210	10.4%
20-39 years	993	49.5%
40-59 years	556	27.7%
60-79 years	235	11.7%
≥80 years	11	0.5%
Marital status		
Married	1,225	61.1%
Unmarried	780	38.9%
Residence		
Rural	1,440	71.8%
Urban	565	28.2%
Family history of psychiatric illness		
Absent	1,803	89.9%
Present	202	10.1%

Psychiatric disorder

Mood disorder was the most common, followed by schizophrenia, schizotypal, and delusional disorders, and neurotic, stress-related, and somatoform disorders (Table 2).

**Table 2:** Psychiatric morbidity according to ICD-10 (N=2,005) ICD-10, International Classification of Diseases 10th Revision

Psychiatric morbidity (ICD-10)		Frequency	Percentage
F00-F09	Organic, including symptomatic, mental disorders	122	6.1%
F10-F19	Mental and behavioral disorders due to psychoactive substance use	190	9.5%
F20-F29	Schizophrenia, schizotypal, and delusional disorders	402	20.0%
F30-F39	Mood (affective) disorders	650	32.4%
F40-F49	Neurotic, stress-related, and somatoform disorder	349	17.4%
F50-F59	Behavioral syndromes associated with physiological disturbances and physical factor	60	3.0%
F60-F69	Disorder of adult personality and behavior	55	2.7%
F70-F79	Mental retardation	45	2.2%
F80-F89	Autism	5	0.2%
F90-F99	Hyperkinetic disorder	15	0.7%
G43-44	Headache syndrome	112	5.6%

Among those patients who had any organic disorder (F00-F09), the most common was dementia (49.2%; 60/122), followed by dementia with depression (21.3%; 26/122), delirium (9.8%; 12/122), and dementia with behavior and psychological symptoms (9.8%; 12/122). Alcohol use disorder was most common (63.2%; 120/190) among substance use disorder (F10-F19), followed by cannabis-induced psychosis (11.1%;21/190) and cannabis use disorder (9.5%; 18/190). Among schizophrenia, schizotypal, and delusional disorders (F20-F29), unspecified psychosis was most common (41.2%; 168/402), followed by acute transient psychotic disorder (29.6; 119/402) and schizophrenia (22.4%; 90/402). Mood disorders were found in majority (32.4%; 650/2005) of the patients. Depression (44.6%; 290/650) was the most common disorder, followed by bipolar affective disorder (40.0%; 260/650); rest were diagnosed with depression with tension-type headache (7.7%; 50/650), psychotic symptoms (6.2%; 40/650), and premature ejaculation (1.5%; 10/650). Anxiety spectrum was most common (65.9%; 230/349) among those who were having neurotic, stress-related, and somatoform disorders, followed by obsessive compulsive disorder (18.6%; 65/349). Among behavioral syndrome associated with psychological disturbance (F50-F59), sleep disorder (66.7%; 40/60) was most commonly seen, followed by post-partum psychosis (25.0%; 15/60) and premature ejaculation (8.3%; 5/60). Borderline personality disorder (63.6%; 35/55) was most commonly seen among adult personality disorder (F60-69). Intellectual disability (66.7%; 30/45) and intellectual disability with behavior impairment (33.3%; 15/45) was contributing toward mental retardation (F70-F79). Among those who were having headache syndrome, majority were having migraine (58.0%; 65/112), and rest were having tension-type headache (42.0%; 47/112).

Association with variables

Organic mental disorder was higher among unmarried individuals compared to married ones (p=0.045), and mental and behavioral disorders due to psychoactive substance use were more common among males than females (p<0.001), with unmarried individuals having a higher frequency than married ones (p=0.001). Mood disorders were more common among females than males (p=0.001), with the highest frequency in individuals aged 20-39 years (p<0.001). Somatoform disorders were more prevalent in females (p=0.004), with the lowest frequency occurring in individuals younger than 20 years (p<0.001). Behavioral syndromes were more frequent in females (p=0.029), with the highest frequency in individuals aged 30-39 years (p=0.015). Adult personality disorder had an equal frequency among males and females (p=0.618), with the highest frequency in individuals aged 30-39 years (p=0.085). Mental retardation had an equal frequency among males and females (p=0.596), with the majority occurring in individuals younger than 40 years (p<0.001). Autism was more common in females, but the difference was insignificant (p=0.707), and all patients were younger than 20 years. Hyperkinetic disorder was more common in males (p=0.833), with the highest frequency occurring in individuals younger than 20 years (p<0.001). Headache syndrome was more common in females (p=0.038), with an insignificant age distribution (p=0.067). Most of the psychiatric disorders were significantly high among the urban population. However, the distribution of psychiatric disorders due to substance abuse and hyperkinetic disorder among rural-urban populations was insignificant.

## Discussion

Maintaining mental well-being is crucial for a good quality of life, but mental illnesses can lead to significant sickness and death globally. The types of psychiatric illness present in a community reflect its disease burden, and understanding their variations is important for creating prevention strategies. This study aimed to examine the pattern of psychiatric illness in a tertiary care hospital in Sagar.

Mood disorder was the most common psychiatric disorder seen among the patients attending the psychiatric OPD, followed by schizophrenia, schizotypal, and delusional disorders, and neurotic stress-related, and somatoform disorders. This is consistent with previous studies that have also reported a high prevalence of mood disorders and schizophrenia [6,7]. However, according to the National Mental Health Survey 2015-16, mental and behavioral disorders caused by psychoactive substance use were the most prevalent in Indians, followed by mood disorder, neurotic and stress-related disorders, and schizophrenia and other psychotic disorders [8]. This variation could be due to differences in the time frame and study type, as well as the use of different rating scales. Studies have consistently found that mood disorders such as depression and bipolar disorder are more prevalent in females than in males [9-12]. This trend is evident across different age groups, with the highest frequency of mood disorders observed among individuals aged 20-39 years. Ferrari et al. found that the prevalence of mood disorders was higher in females than in males, and this difference was consistent across different regions and cultures [12]. The reasons for this gender difference are complex and likely involve a combination of biological, psychological, and social factors [13].

Our results showed that substance use disorders are more common among unmarried males, which was consistent with previous studies [11,14]. Studies have consistently reported that males have a higher prevalence of substance use disorders compared to females [8,11,14]. The study found that somatoform disorders were more prevalent in females, especially among those younger than 20 years. This was consistent with previous research that reported higher rates of somatoform disorders among females [11,15]. The study found that behavioral syndromes were more frequent in females, particularly in individuals aged 30-39 years. This was consistent with previous research that reported gender and age differences in the prevalence of behavioral syndromes and higher prevalence of behavioral disorders in women compared to men [11].

Autism was more common in females, but the difference was insignificant, and all patients were under 20 years old. This finding was inconsistent with previous studies showing that autism was more common in males than in females [16]. Hyperkinetic disorder was more common in males, with the highest frequency occurring in individuals younger than 20 years. This finding was consistent with previous studies showing that hyperkinetic disorder (also known as attention deficit hyperactivity disorder) was more common in males than in females [17]. Similar to the previous study, headache syndrome was more common in females, with an insignificant age distribution [18].

Our results have revealed that the prevalence of psychiatric disorders was generally higher in urban areas compared to rural areas, except for disorders related to substance abuse and hyperactivity, where the distribution was not significantly different. The process of urbanization has also been linked to an increased risk of certain psychiatric disorders such as schizophrenia, other psychotic disorders, anxiety, and mood disorders [19,20]. However, the relationship between psychiatric disorders and urban or rural living was not always consistent and varied depending on the study [14,20].

This study’s strengths include its large sample size, which allowed for a more comprehensive understanding of psychiatric morbidity in a tertiary care center. Its retrospective design and single institutional-based nature may have led to biases and limited generalizability. This hospital record-based design also restricted detailed information collection on various factors, such as socio-economic status, medical comorbidity, and treatment details.

## Conclusions

Our study sheds light on the types of psychiatric disorders among patients seeking treatment at a tertiary care center. The results can aid clinicians in providing better care and emphasize the need for early detection and treatment of mental illnesses. However, to gain a more comprehensive understanding of psychiatric illness, additional research is needed in community-based settings, focusing on clinical profiles, attitudes of patients and families toward psychiatric illness, and treatment burden.
